# Vaccination—The Royal Commission

**Published:** 1897-04

**Authors:** M. R. Leverson


### VACCINATION.—THE ROYAL COMMISSION.

EDITOR OF THE HOMEOPATHIC PHYSICIAN.

DEAR SIR:—I beg your readers to make the following corrections at p. 84 of your March number in paragraph 3, line 4: "Answer" should be "answers," "Q." should be "Qus," after "1,255" insert "and 1,415," after the parenthesis insert, "See also Q. 1,377." So that the line would now read, ——registered." (Compare answers to Qus. 1,225 and 1,415, *supra*, p. 81.) See also answer to Q. 1,377, "We, etc.

In reply to your observations supplementing my article on page 108 of the same issue of THE HOMEOPATHIC PHYSICIAN, I desire to state that for most of the statements made, I refer to the authorities given in my paper on "Vaccination, Should It be Enforced by Law." With regard to its easy and rapid cure, and to its non-infectiousness when properly treated, I will state that I hope to be able to present to the International Homeopathic Society, at its meeting at Niagara in June next, the clinical history of one out of thirty cases treated by me during the past three years, together with an account of a "proving" of the bath recommended by Mr. Pickering, which I made *before* I ventured to assay his plan. Till then I pray your patience.

Faithfully yours,

M. R. LEVERSON.

